# Developmental coordination disorder, psychopathology and IQ in 22q11.2 deletion syndrome

**DOI:** 10.1192/bjp.2017.6

**Published:** 2018-01

**Authors:** Adam C. Cunningham, Sue Delport, Wendy Cumines, Monica Busse, David E. J. Linden, Jeremy Hall, Michael J. Owen, Marianne B. M. van den Bree

**Affiliations:** 1 MRC Centre for Neuropsychiatric Genetics and Genomics, Division of Psychological Medicine and Clinical Neurosciences, Cardiff University School of Medicine, Cardiff, UK; 2 School of Healthcare Sciences, Cardiff University, Cardiff, UK; 3 Centre for Trials Research, Cardiff University, Cardiff, UK; 4 MRC Centre for Neuropsychiatric Genetics and Genomics, Division of Psychological Medicine and Clinical Neurosciences, Cardiff University School of Medicine, Cardiff, UK

## Abstract

**Background:**

22q11.2 deletion syndrome (22q11.2DS) is associated with high rates of neurodevelopmental disorder, however, the links between developmental coordination disorder (DCD), intellectual function and psychiatric disorder remain unexplored.

**Aims:**

To establish the prevalence of indicative DCD in children with 22q11.2DS and examine associations with IQ, neurocognition and psychopathology.

**Method:**

Neurocognitive assessments and psychiatric interviews of 70 children with 22q11.2DS (mean age 11.2, s.d. = 2.2) and 32 control siblings (mean age 11.5, s.d. = 2.1) were carried out in their homes. Nine children with 22q11.2DS and indicative DCD were subsequently assessed in an occupational therapy clinic.

**Results:**

Indicative DCD was found in 57 (81.4%) children with 22q11.2DS compared with 2 (6.3%) control siblings (odds ratio (OR) = 36.7, *P* < 0.001). Eight of nine (89%) children with indicative DCD met DSM-5 criteria for DCD. Poorer coordination was associated with increased numbers of anxiety, (*P* < 0.001), attention-deficit hyperactivity disorder (ADHD) (*P* < 0.001) and autism-spectrum disorder (ASD) symptoms (*P* < 0.001) in children with 22q11.2DS. Furthermore, 100% of children with 22q11.2DS and ADHD had indicative DCD (20 of 20), as did 90% of children with anxiety disorder (17 of 19) and 96% of children who screened positive for ASD (22 of 23). The Developmental Coordination Disorder Questionnaire score was related to sustained attention (*P* = 0.006), even after history of epileptic fits (*P* = 0.006) and heart problems (*P* = 0.009) was taken into account.

**Conclusions:**

Clinicians should be aware of the high risk of coordination difficulties in children with 22q11.2DS and its association with risk of mental disorder and specific neurocognitive deficits.

**Declaration of interest:**

None.

Motor coordination problems can seriously affect a child's life, including activities of daily living (e.g. eating, dressing, grooming), self-esteem, pastime activities, social relationships and academic attainment.^1^ Motor dysfunction can furthermore compound risk for psychopathology,^2^ even into adulthood.^3^ Developmental coordination disorder (DCD) is a neurodevelopmental disorder characterised by motor function that is markedly deficient given the person's chronological age and measured intelligence, that is not explained by any overt motor or sensory deficit.^4^ DCD is often comorbid with other neurodevelopmental disorders, particularly attention-deficit hyperactivity disorder (ADHD), but also anxiety disorders and autism-spectrum disorder (ASD).^2^^,^^5^^–^^7^ For example, up to 50% of children with DCD have been reported to have a diagnosis of ADHD, primarily of the inattentive subtype.^8^ DCD is also associated with neurocognitive deficits, including in executive functioning.^9^ The relationship between IQ and DCD has not been rigorously tested as, because of the current definition, most studies of DCD have excluded individuals with an IQ in the borderline or intellectual disability range. However, a better understanding of the presence of motor dysfunction in children with intellectual disability can pave the way towards suitable interventions and can also shed light on possible shared underlying processes.

A diagnosis of DCD is established through a series of multiple assessments that establish coordination and the impact of difficulties on daily life. Screening tools, such as the Developmental Coordination Disorder Questionnaire (DCDQ), can be used effectively in large populations, and have been shown to have good agreement with more detailed movement assessments in populations with attention difficulties, intellectual difficulties or healthy children.^10^^,^^11^ However, there are no studies comparing the validity of the DCDQ as a measure of coordination in populations with chromosomal disorders.

22q11.2 deletion syndrome (22q11.2DS) is a rare chromosomal disorder that affects ≈1 in 2000–4000 live births and is caused by a hemizygous microdeletion on the long arm of chromosome 22.^12^ In childhood, the deletion is associated with high rates of ADHD, ASD, anxiety disorders and oppositional defiant disorder.^13^^,^^14^ 22q11.2DS is also one of the strongest known biological risk factors for the development of schizophrenia, with about 25% of adult patients with 22q11.2DS affected.^15^ Mild or moderate intellectual disability is common, with reports that the mean IQ of patients with 22q11.2DS is approximately 30 points lower than that of unaffected siblings.^13^^,^^14^ Coordination problems are increasingly recognised as a feature of 22q11.2DS, particularly problems with balance, bimanual coordination and visuomotor skills, that may be independent of IQ.^16^^–^^20^ Moreover, 22q11.2DS is associated with a number of factors that have been associated with motor problems such as preterm birth, hypotonia, seizures and abnormalities of the central nervous system.^21^ We are not aware of studies that have investigated the prevalence of DCD, or of a clear pathway to assigning a diagnosis of DCD in this population. Furthermore, there are no papers on the links between coordination problems and IQ and other neurocognitive function or psychopathology in carriers of this deletion. It may be the case that DCD may index a greater overall neurodevelopmental difficulty, and therefore be related to other psychopathology.

We set out to address these gaps in the literature. Our first aim was to investigate the prevalence of indicative DCD in children with 22q11.2DS in comparison with siblings without the deletion by screening for DCD using the DCDQ. Our second aim was to evaluate the degree to which the DCDQ captured the motor performance difficulties associated with DCD in this population. This was achieved by conducting the gold-standard Movement Assessment Battery for Children-2 (MABC-2)^22^ in an occupational therapy clinic in a subset of nine of the children with indicative DCD, to ensure that the DCDQ is accurately reflecting coordination difficulties in this population. Third, we investigated the relationships between indicative DCD and psychiatric problems that are common in children with 22q11.2DS (ADHD, ASD and anxiety disorder). Fourth, we aimed to explore the relationship between indicative DCD and IQ and specific neurocognitive functions in this population. We hypothesised that there would be a high incidence of indicative DCD in children with 22q11.2DS and that most of the children who screened positive would meet diagnostic criteria for DCD; and furthermore, that indicative DCD would be associated with risk of ADHD, ASD and anxiety disorder, as well as poorer cognitive ability. Finally, we also explored whether specific medical aspects of 22q11.2DS (preterm birth, history of heart problems and epileptic fits) contributed to risk of indicative DCD and possible links with psychopathology and cognition.

## Method

### Participants

Participants were members of the ongoing Experiences of CHildren with cOpy number variants (ECHO) study. Children with 22q11.2DS were recruited through medical genetics clinics across the UK, charities for chromosomal conditions as well as 22q11.2DS specifically (Unique, 22Crew and MaxAppeal!) and word of mouth. Unaffected siblings closest in age to the child with the deletion were also invited to take part. Inclusion criteria were age 6 years of age or older (in order for the psychiatric assessment to be valid) and for the child with the deletion, confirmation of 22q11.2DS by Medical Genetics laboratories, using standard methods (fluorescence *in situ* hybridisation/microarray) and subsequently in the laboratory of the MRC Centre for Neuropsychiatric Genetics and Genomics at Cardiff University, using microarray techniques. The current study was based on 70 children with 22q11.2DS (58.6% male, mean age 11.2 years, s.d. = 2.2) and 32 unaffected siblings (43.8% male, mean age 11.5, s.d. = 2.1). Children with 22q11.2DS did not differ in age (*P* = 0.50) or gender distribution (*P* = 0.16) from the control siblings. Informed and written consent was obtained prior to recruitment from the carers of the children and recruitment was carried out in agreement with protocols approved by the appropriate research and National Health Service ethics and research and development committees. The primary carers of the children provided information on the children's physical health. Ten children (14.2%) with 22q11.2DS were born earlier than 37 weeks, 12 (17.1%) had a history of epileptic fits, 34 (48.6%) of a heart problem and 2 (2.9%) of low calcium levels in early childhood. None of the children were receiving medication for ADHD and one child was taking sodium valproate for epilepsy, along with fluoxetine and risperidone for a psychotic disorder.

### Coordination assessment (DCDQ/MABC-2)

The DCDQ^23^ was completed by the primary carer. It is designed to screen for motor coordination impairments in children 5–15 years old and is well validated.^10^ DCDQ scores range from 15 to 75, with discrimination thresholds that are dependent on age. In general, lower scores indicate greater coordination problems. The DCDQ assesses coordination either while moving or when using the hands. It yields a total score as well as separate scores for three subscales: control during movement, fine motor/handwriting and general coordination scores. Participants were categorised into those with and without indicative DCD based on DCDQ total score compared with the appropriate age threshold.

The DCDQ can be used to indicate whether a child is likely to have DCD, although additional assessments are necessary to establish the diagnosis. The DCDQ is useful in establishing functional difficulties in everyday life because of coordination impairment, but reliable and valid motor assessments are required to accurately assess if motor performance is substantially below the level expected given the child's chronological age and/or IQ. To establish the extent to which the DCDQ captures DCD in this population, a subsample of nine children with 22q11.2DS (mean age 12.05, s.d. = 2.56, 6 (67%) male) who screened positive on the DCDQ were assessed by trained and experienced occupational therapists using the MABC-2.^22^ As the ECHO study recruits children with 22q11.2DS from all over the UK, not all families are able to travel to Cardiff for assessments. Therefore, these nine families were selected based on proximity to Cardiff University. Using the MABC-2, we obtained an overall score, and scores for the three subdomains: manual dexterity, aiming/catching and balance. Scores on the MABC-2 below the fifth percentile generally indicate severe problems with motor coordination that require intervention. Information from the MABC-2, and previously collected assessments were used to establish a research diagnosis of DCD based on DSM-5 criteria.^24^

Parents also completed questions on three developmental milestones: age at which the child learned to ride a bike, do up their shoelaces and fasten buttons. These milestones give a general measure of gross and fine motor skill development, complementing other information. Sample sizes for milestone comparisons differ as only a proportion of participants had attained the milestones at the time of data collection.

### IQ assessment

Full-scale, verbal and performance IQ was obtained by administering the Wechsler Abbreviated Scale of Intelligence (WASI).^25^

### Cognitive function assessment

Cognitive function was assessed using tasks that are relevant for risk of psychopathology. Processing speed (five- choice reaction time task), sustained attention (rapid visual processing task), spatial working memory (SWM), spatial planning (stockings of Cambridge) and visual attention (match to sample task) were assessed using the Cambridge Neuropsychological Test Automated Battery (CANTAB).^26^ Furthermore, the Wisconsin Card Sorting Test 32 (WCST)^27^ was also administered, where the number of perseverative errors measures the set shifting ability of executive function. Non-perseverative errors are also reported. All neurocognitive measures were standardised to have a mean of zero and a standard deviation of one, with the exception of IQ.

### Psychopathology assessment

The Social Communication Questionnaire (SCQ),^28^ was used to screen for ASD symptoms. Total scores can range from 0 to 39, and a score of 15 or greater is suggestive of putative ASD. The SCQ yields a total score and three subscale scores (behaviour, social and communication). The behaviour subscale measures repetitive and stereotyped behaviours, the social scale probes aspects of reciprocal social interaction such as eye gaze and social smiling, and the communication subscale asks about communication ability including social chat and gestures.

The semi-structured Child and Adolescent Psychiatric Assessment interview (CAPA)^29^ was conducted by trained psychologists with the primary caregiver and children themselves where possible and appropriate. Interviews were audiotaped and DSM-5 diagnosis obtained during consensus meetings lead by a child and adolescent psychiatrist. We did not consider diagnoses to be mutually exclusive. Other psychiatric diagnoses and symptoms were obtained from the CAPA. A symptom was counted as present if the individual had scored a two or three on the relevant CAPA question. Anxiety symptoms included any symptom of generalised anxiety disorder, social phobia, specific phobia, separation anxiety, panic disorder with and without agoraphobia, agoraphobia and obsessive-compulsive disorder. An individual with a research diagnosis of one of these anxiety disorders was classified as having ‘any anxiety disorder’.

All assessments were carried out as part of the ongoing ECHO study, either in participants' homes or during visits to our laboratory at Cardiff University. Sample sizes for analyses using the IQ, cognitive and symptom data differ, as complete data-sets were not available for some participants, because of the individuals having difficulties in completing measures.

### Statistical analysis

Statistical analysis was carried out in R version 3.3.3 (https://www.R-project.org/). Differences in group statistics scores between the 22q11.2DS and sibling groups were established using *t*-tests or Wilcoxon tests where appropriate with respect to normality. DCD and mental disorder prevalence in children with 22q11.2DS compared with control siblings was examined using a chi-squared test. Spearman correlations were used to assess associations between the DCDQ total score and age of attaining milestones. Pearson correlation was used to test association between indicative DCD and MABC-2 score. Associations between psychiatric symptoms (ADHD, SCQ score, any anxiety disorder), IQ and neurocognitive measures and indicative DCD were established using linear regression. Predictors were entered hierarchically, age first, then gender and finally the psychopathology, cognition or IQ variable. Sensitivity analyses were carried out to investigate whether comorbid factors (preterm birth, history of epileptic fits or heart problems) contributed to our findings. We calculated rates of indicative DCD excluding the children with either preterm birth, or epileptic fits or heart problems. Furthermore, we repeated the regression analyses including these conditions as covariates one at a time. As levels of medication use (1.4%, 1/70) and history of hypocalcaemia (2.8%, 2/70) were low, these were not taken into consideration in these analyses.

## Results

Descriptive statistics about the families are presented in Table 1.

**Table 1:** Descriptive statistics of sample

Characteristics	Variable	*n*	χ^2^ *(P)*
Mother's ethnic background, *n* (%)		75	
European	69 (92.0)		
Mixed	5 (6.7)		
Unknown	1 (1.3)		

Inheritance, *n* (%)		70	
*De novo*	58 (82.9)		
Inherited	6 (8.6)		
Unknown	6 (8.6)		

Highest maternal qualification, *n* (%)		75	
High (university degree and/or other higher postgraduate qualification)	18 (24.0)		
Middle (A-levels/highers/vocational training)	34 (45.3)		
Low (O-levels/GCSEs)	15 (20.0)		
No school leaving exams	8 (10.7)		

Family Income, *n* (%)		75	
≤£19 999	19 (25.3)		
£20 000 - £39 999	22 (29.3)		
£40 000 - £59 999	16 (21.3)		
≥£60 000	15 (20.0)		
Unknown	3 (4.0)		

Age, years: mean (s.d.) range			
Probands		70	
Male	11.10 (2.20) 6.20–14.87		
Female	11.58 (2.31) 7.11–14.75		
Siblings		32	
Male	11.75 (1.58) 9.24–14.89		
Female	11.39 (2.40) 6.18–14.88		

Gender, *n* (%)			1.940 (0.163)
Probands		70	
Male	41 (58.6)		
Female	29 (41.4)		
Siblings		32	
Males	14 (43.8)		
Female	18 (56.3)		

### Prevalence of indicative DCD in 22q11.2DS

Children with 22q11.2DS had lower scores on the DCDQ (22q11.2DS group median 39.5; controls median 73.5, *P* < 0.001) and all subscales (control during movement *P* < 0.001, fine motor *P* < 0.001, general coordination *P* < 0.001), reflecting poorer coordination. In total 57 children with 22q11.2DS met criteria for indicative DCD (81.4%) compared with two control siblings (6.3%) (χ^2^ = 50.9, *P* < 0.001, odds ratio (OR) = 36.7). Similar numbers of males and females (*n* = 36, 87.8% of males, *n* = 21, 72.4% of females) with 22q11.2DS met criteria for indicative DCD (χ^2^ = 2.66, *P* = 0.103, OR = 2.05). Males had a median score of 36 on the DCDQ *v.* 45 in females (*P* = 0.013).

Children with 22q11.2DS had a higher mean age of learning to ride a bike and do up buttons compared with control siblings (difference of 14.26 months for learning to ride a bike; 22.21 months for doing up buttons, Table 2). Developmental coordination problems correlated with age of attainment of doing up buttons (*r* = −0.51, *P* < 0.001); but not tying shoelaces (*r* = −0.43, *P* = 0.060) or riding a bike (*r* = −0.27, *P* = 0.086); whereas no associations were found for the siblings.

**Table 2:** Results of group comparisons

Measure	22q11.2 DS group	Control siblings	Total,^a^ *n*		χ^2^	OR	*t*	*z*	95% CI for differences in means/medians	*P*
			22q11.2 DS group	Control siblings						
Indicative developmental coordination disorder, *n*			70	32	50.9	36.7				<0.001
Positive	57	2								
Negative	13	30								

Age motor milestones achieved, months:^a^ mean (s.d.)										
Learned to ride a bike	75.88 (20.45)	61.62 (18.66)	40	27			2.95		(4.58 to 23.91)	0.005
Learned to do up buttons	72.21 (24.74)	50.00 (14.83)	48	25			4.78		(12.95 to 31.47)	<0.001
Learned to tie shoelaces	95.45 (29.97)	82.17 (22.7)	20	24			1.63		(−3.26 to 29.83)	0.112

Psychopathology, median (IQR)										
Attention–deficit hyperactivity disorder, symptom count	5 (7.75)	0 (0.00)	70	30				5.87	(3 to 7)	<0.001
Putative autism-spectrum disorder score	11 (11.00)	1 (2.00)	67	32				6.94	(7 to 12)	<0.001
Anxiety symptoms	3 (9.75)	0 (1.00)	66	29				3.79	(1 to 3)	<0.001

IQ, mean (s.d.)										
Full-scale IQ	70.75 (11.94)	104.58 (15.71)	70	31			−10.65		(−40.22 to −27.44)	<0.001
Performance IQ	74.55 (12.87)	102.68 (17.46)	67	31			−8.02		(−35.19 to −21.06)	<0.001
Verbal IQ	70.97 (12.73)	105.58 (15.68)	68	31			−10.78		(−41.07 to −28.15)	<0.001

Cognitive processing,^b^ mean (s.d.)										
Processing speed (five-choice reaction time)	−0.18 (1.16)	0.36 (0.38)	61	31			−3.30		(−0.86 to −0.21)	0.001
Sustained attention (rapid visual processing)	−0.26 (1.10)	0.47 (0.56)	55	30			−4.04		(−1.08 to −0.37)	<0.001
Visual attention (match to sample)	−0.23 (1.03)	0.45 (0.61)	60	31			−3.81		(−1.03 to −0.32)	<0.001
Spatial planning	−0.27 (0.99)	0.52 (0.81)	57	29			−3.96		(−1.19 to −0.39)	<0.001
Spatial working memory	−0.34 (0.92)	0.72 (0.77)	67	31			−5.96		(−1.41 to −0.71)	<0.001
Set shifting ability	0.06 (0.82)	−0.13 (1.32)	66	30			0.76		(−0.33 to 0.73)	0.450
Errors on the Wisconsin Card Sorting Task	−0.32 (0.99)	0.70 (0.60)	66	30			−6.25		(−1.34 to −0.70)	<0.001

22q11.2 DS, 22q11.2 deletion syndrome; IQR, interquartile range.

a. Total *n* given for age motor milestone achieved is those who had achieve this milestone at the time of data collection.

b. For spatial planning: stockings of Cambridge – problems solved in minimum moves; set shifting ability: perseverative errors on the Wisconsin Card Sorting Task (WCST); errors on the WCST: non-perseverative errors.

Of the nine children with 22q11.2DS assessed with the MABC-2 eight had overall scores below the fifth percentile. Of the nine, three had scores below the fifth percentile on the aiming/catching domain, four had scores below the fifth percentile in the manual dexterity domain and five had scores below the fifth percentile in the balance domain. Performance on the individual domains was variable, however, children did not always score below the fifth percentile in all domains, rather, they frequently performed markedly poorly in one or two domains and slightly better in the third domain. Of the eight children who had overall scores below the fifth percentile, three scored below the fifth percentile in one domain, three in two domains and one in three domains. DCDQ total score was not associated with MABC-2 overall standard score, (*r* = 0.63, *P* = 0.070).

### Associations between indicative DCD and psychopathology

In total, 32.9% (23/70) of children with 22q11.2DS met criteria for ADHD, compared with 3.3% (1/30) of siblings; 29.0% (20/69) of children with 22q11.2DS met criteria for any anxiety disorder, compared with 6.7% (2/30) of siblings and 34.3% (23/67) of children with 22q11.2DS screened positive for putative ASD whereas no siblings did (0/32). Similarly, the rates of ADHD, putative ASD and anxiety symptoms were higher in children with 22q11.2DS than siblings (Table 2).

**Fig. 1:**
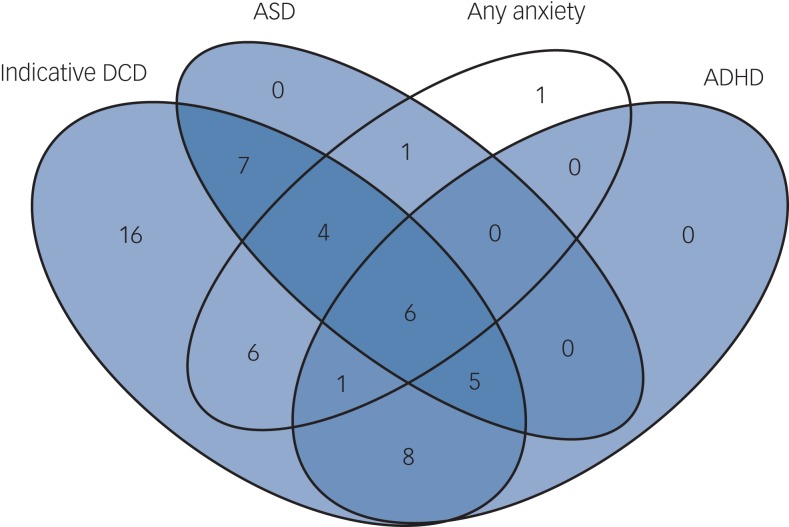
Comorbidity in the participants with 22q11.2 deletion syndrome.

ASD, autism-spectrum disorder; ADHD, attention-deficit hyperactivity disorder; DCD, developmental coordination disorder.

Of the children with indicative DCD and complete diagnosis data for ASD, ADHD and anxiety, 69.8% (37/53) had at least one psychiatric disorder compared with 15.4% (2/13) of individuals without indicative DCD (*P* < 0.001, OR = 5.84). Fig. 1 shows the high rate of co-occurrence between motor dysfunction and psychopathology. In total, 30.2% (16/53) of individuals with indicative DCD met criteria for at least two, and 11.3% (6/53) for all three disorders. Of children with 22q11.2DS and indicative DCD, 38% (20/53) met the criteria for ADHD, compared with 0% (0/13) of children without indicative DCD (*P* = 0.008, OR = 3.47). Percentages for putative ASD were 41.5% (22/53) *v.* 7.7% (1/13) (*P* = 0.022, OR = 2.46) and for anxiety disorder 32.1% (17/53) *v.* 15.4% (2/13) (*P* = 0.234, OR = 1.7) in children with and without indicative DCD, respectively. All (100%, 20/20) children with ADHD had indicative DCD, as did 89.5% (17/19) of children with any anxiety disorder and 95.7% (22/23) of children with putative ASD.

The DCDQ total score was associated with ADHD symptom count (*P* < 0.001), but not age (*P* = 0.497) or gender (*P* = 0.374) and this association was driven by inattentive (*P* < 0.001) but not hyperactivity symptoms (*P* = 0.051). Scores on all three subscales of the DCDQ were associated with ADHD symptoms (fine motor skill *P* < 0.001, control during movement *P* < 0.001, general coordination *P* < 0.001).

DCDQ total score was also associated with putative ASD score (*P* < 0.001), but not age (*P* = 0.304) or gender (*P* = 0.188). The control during movement (*P* < 0.001), general coordination (*P* = 0.003), as well as fine motor (*P* < 0.001) subscales were all associated with putative ASD score. Further analysis showed that all three subtests of the putative ASD score (behaviour *P* < 0.001, communication *P* < 0.001, social *P* = 0.026) predicted DCDQ total score.

DCDQ total score was furthermore associated with anxiety symptoms (*P* < 0.001), along with gender (*P* = 0.041), with boys having lower DCDQ scores, but not age (*P* = 0.341). Scores on the fine motor skill subscale were associated with anxiety symptoms (*P* = 0.004) and gender (*P* = 0.005). The scores on the general coordination subscale were also associated with anxiety symptoms (*P* = 0.001) and gender (*P* = 0.033). Anxiety symptoms (*P* = 0.004) but not gender predicted the control during movement score.

### Association between indicative DCD and IQ

Mean full-scale IQ of the siblings was higher than in children with 22q11.2DS (Table 2). Of the children with 22q11.2DS with data for full data for IQ (*n* = 67), 4 (5.97%) had moderate intellectual disability (IQ < 55), 29 (43.3%) mild intellectual disability (IQ 55–70), 24 (35.8%) had an IQ in the borderline range (71–85) and 10 (14.9%) an average IQ (86–115). This is in comparison with one (3.1%) sibling with mild intellectual disability. Indicative DCD was associated with full-scale IQ in children with 22q11.2DS (*P* = 0.038).

### Relationships between indicative DCD and cognitive tasks

Children with 22q11.2DS performed more poorly than siblings on the processing speed, sustained attention, visual attention, spatial planning and executive functioning tasks (Table 2). In children with 22q11.2DS, DCDQ score was not related to set shifting ability (perseverative errors *P* = 0.444), or total errors (non-perseverative errors *P* = 0.449) on the WCST, nor the processing speed (reaction time *P* = 0.424), spatial planning (problems solved in minimum moves *P* = 0.765), and SWM (number of errors, *P* = 0.733) tasks of the CANTAB. However, an association was found with poorer performance on visual (*P* = 0.038) as well as sustained attention (*P* = 0.006).

### Sensitivity analysis of comorbid medical aspects

Excluding children with 22q11.2DS with preterm birth, a history of heart problems, or epileptic fits did not affect the rates of indicated DCD, nor any of the links between the DCDQ and psychiatric disorder. However, with regards to neurocognitive function, including epileptic fits or heart problems as a covariate in the analysis reduced the associations between DCDQ score and both full-scale IQ (*P* = 0.056 or *P* = 0.062, respectively) as well as visual attention (*P* = 0.062 and *P* = 0.055, respectively), but not sustained attention (*P* = 0.006 and *P* = 0.009, respectively). Preterm birth did not affect the associations between DCDQ score and the neurocognitive measures.

## Discussion

### DCD prevalence

The findings indicate that serious motor coordination problems are common in 22q11.2DS, with over 80% of our sample of deletion carriers meeting criteria for indicative DCD. Furthermore, indicative DCD indexed risk of ADHD, ASD and anxiety disorder as well as poorer sustained attention. The prevalence of indicative DCD in our sample differed between males and females with 22q11.2DS, conforming to the pattern of the male preponderance reported in the general population.^30^ This contrasts with other disorders such as ADHD, where the prevalence seems to be equal between the genders in 22q11.2DS.^14^ DCDQ total score was correlated with age of attainment of developmental milestones providing further support for the validity of the DCDQ in this population. Furthermore, 88.9% of children (eight of nine) who had diagnostic assessments in the occupational therapy clinic, using the gold-standard MABC-2, met criteria for DCD. This provides additional support for using the DCDQ as a proxy measure of developmental coordination dysfunction. Scores on the MABC-2 below the fifth percentile generally indicate problems with motor coordination that require intervention. However, performance on individual domains of the MABC-2 was variable, with no single domain emerging as consistently affected. As such, this evidence suggests that much like the psychiatric and cognitive phenotype in 22q11.2DS, there is also considerable variability in the motor phenotype.

### Psychopathology

The majority of children with indicative DCD (70%) were found to have at least one psychiatric disorder, including high rates of ADHD, anxiety disorder and ASD symptoms. Indicative DCD was found to be related to ADHD, with children with more inattentive symptoms having greater difficulties with motor coordination. Studies in children with ADHD not selected for the presence of a copy number variant have indicated they are more likely to have impairments in both gross and fine motor skills, particularly if the child has ADHD of the inattentive subtype. Our previously published work comparing children with ADHD with and without 22q11.2DS has found that the deletion is associated with a considerably higher rate of the inattentive subtype as well as a lower rate of hyperactive-impulsive symptoms. We have suggested this may contribute to underdiagnosis of ADHD in children with the deletion.^31^ We also found that children with 22q11.2DS and higher numbers of ASD symptoms had poorer coordination, a finding that is similar to studies of children with DCD not selected for a chromosomal disorder.^32^ Our finding that children with indicative DCD had higher levels of anxiety symptoms is in line with other research showing links between anxiety and DCD.^2^ Excessive worry is a well-documented phenomenon in 22q11.2DS,^13^^,^^14^ however, it is not clear whether anxiety and DCD share biological pathways, or whether DCD contributes to anxiety as a result of worries about performance. Such worries may particularly affect children in social settings, for fear of humiliation and social exclusion.^2^ Future longitudinal studies investigating the developmental links between motor function and psychopathology can contribute to better understanding of these issues.

### DCD and IQ and other neurocognitive functions

Indicative DCD was related to IQ in children with 22q11.2DS. This suggests that the observed coordination difficulties seen in this population can be partially explained by a general deficit in IQ. This agrees with studies of children with DCD not selected for having a chromosomal disorder^9^ and suggests that within an intellectually disabled population, level of impairment is associated with motor dysfunction. This is in contrast to previous reports by our group on IQ and psychopathology in children with 22q11.2DS,^13^ where we found no association.

Indicative DCD was also related to visual attention ability as measured by the match to sample task and sustained attention as measured by the rapid visual processing task of the CANTAB. Together with the here reported association between motor coordination difficulties and the inattentive subtype of ADHD, this suggests common processes underlying coordination and attention. However, it is unclear if coordination is impaired as a result of an inability to direct attention appropriately, or if the same brain processes are required for good coordination and attention.

The associations between DCDQ score and visual attention were reduced, however, when epileptic fits or a history of heart problems were included in the analysis as covariates. This indicates that other medical aspects associated with 22q11.2DS can contribute to specific interrelationships and underlines the complex aetiology of the condition. The link between sustained attention and the DCDQ remained, however, unchanged by these medical aspects. Impairments in sustained attention^33^ as well as motor coordination^34^ have previously been reported in schizophrenia. The current sample is too young to investigate the links with schizophrenia risk; however, this represents an interesting avenue for future research.

### Strengths and limitations

To our knowledge, this is the first study to examine the prevalence of DCD and its relationship with IQ, and other neurocognition and neurodevelopmental symptoms in 22q11.2DS. The relatively large sample and availability of sibling controls for comparisons are additional strengths. Also, the availability of medical information allowed us to conduct sensitivity analysis and show that the high rates of indicated DCD could not be explained by preterm birth, a history of epileptic fits or heart problems. A limitation was the small subset of individuals evaluated using the MABC-2.

As most coordination and psychopathology data was collected through home visits a full neurological assessment was not possible, therefore we cannot rule out other contributing conditions such as hyper-/hypotonia that can affect coordination. Finally, both the DCDQ and MABC-2 are measures of overall coordination, and do not allow insights into underlying sensorimotor and visual information processing deficits.

### Theoretical implications

The high rate of indicative DCD in 22q11.2DS is a novel finding and our occupational therapy assessments in a subsample indicate that the majority of children who screen positive do meet diagnostic criteria for DCD. The presence of coordination deficits raises the question of the changes in neural substrates that result from 22q11.2 deletion. The coordination deficits may be because of disruption of the cerebellum, which has been implicated in both motor and cognitive syndromes,^35^ and shows consistent abnormalities in 22q11.2DS.^36^^,^^37^ Cerebellar dysfunction has also been repeatedly observed in neurodevelopmental disorders, including ASD and ADHD. Other biological mechanisms that could be involved include striatal dysfunction, as increased volume of the striatum^38^^–^^40^ and calcification of the basal ganglia^40^^,^^41^ have been observed in 22q11.2DS. In addition, the 22q11.2 deletion is associated with early-onset Parkinsonism.^42^^,^^43^ The high comorbidity between anxiety disorder, ADHD and ASD may also point towards common neural disruptions. The precise origin of the coordination impairments is not yet known and it is unclear whether motor coordination problems are a common feature of other copy number variant disorders (for example, duplication of 22q11.2, or deletion/duplication of 1q21.1 or 16p11.2). More generally, coordination difficulties may index a general neurodevelopmental impairment in frontostriatal and related circuitry that may reflect risk of other psychopathologies. Future studies should use detailed assessment of fundamental motor control processes, using kinematic assessment, for example. This would allow investigation of deficits of these fundamental processes and may help identify a cause of coordination difficulties.

### Clinical implications

The immediate clinical implication of our findings is that there should be an increased vigilance for motor impairments in children with 22q11.2DS so that appropriate support measures can be introduced as early as possible. In addition, DCD is not usually diagnosed or considered in those with intellectual disability, as the motor deficit must be demonstrated to be in excess of what would be expected for a given IQ, but our findings indicate that the majority of children in this study are affected by potentially serious motor problems. A formal diagnosis of DCD may facilitate access to appropriate support and interventions.

## References

[1] Cantell MH, Smyth MM, Ahonen TP. Clumsiness in adolescence: educational, motor, and social outcomes of motor delay detected at 5 years. Adapt Phys Act Q 1994; 11: 115–29.

[2] Pratt ML, Hill EL. Anxiety profiles in children with and without developmental coordination disorder. Res Dev Disabil 2011; 32: 1253–9. 21377831 10.1016/j.ridd.2011.02.006

[3] Kirby A, Williams N, Thomas M, Hill EL. Self-reported mood, general health, wellbeing and employment status in adults with suspected DCD. Res Dev Disabil 2013; 34: 1357–64. 23417140 10.1016/j.ridd.2013.01.003

[4] Zwicker JG, Missiuna C, Harris SR, Boyd LA. Developmental coordination disorder: a review and update. Eur J Paediatr Neurol 2012; 16: 573–81. 22705270 10.1016/j.ejpn.2012.05.005

[5] Dziuk MA, Gidley Larson JC, Apostu A, Mahone EM, Denckla MB, Mostofsky SH. Dyspraxia in autism: association with motor, social, and communicative deficits. Dev Med Child Neurol 2007; 49: 734–9. 17880641 10.1111/j.1469-8749.2007.00734.x

[6] Kadesjö B, Gillberg C. Attention deficits and clumsiness in Swedish 7-year-old children. Dev Med Child Neurol 1998; 40: 796–804. 9881675 10.1111/j.1469-8749.1998.tb12356.x

[7] Kopp S, Beckung E, Gillberg C. Developmental coordination disorder and other motor control problems in girls with autism spectrum disorder and/or attention-deficit/hyperactivity disorder. Res Dev Disabil 2010; 31: 350–61. 19910158 10.1016/j.ridd.2009.09.017

[8] Kadesjö B, Gillberg C. Developmental coordination disorder in Swedish 7-year-old children. J Am Acad Child Adolesc Psychiatry 1999; 38: 820–8. 10405499 10.1097/00004583-199907000-00011

[9] Wilson PH, Ruddock S, Smits-Engelsman B, Polatajko H, Blank R. Understanding performance deficits in developmental coordination disorder: a meta-analysis of recent research. Dev Med Child Neurol 2013; 55: 217–28. 23106668 10.1111/j.1469-8749.2012.04436.x

[10] Wilson BN, Crawford SG, Green D, Roberts G, Aylott A, Kaplan BJ. Psychometric properties of the revised Developmental Coordination Disorder Questionnaire. Phys Occup Ther Pediatr 2009; 29: 182–202. 19401931 10.1080/01942630902784761

[11] Wilson BN, Kaplan BJ, Crawford SG, Campbell A, Dewey D. Reliability and validity of a parent questionnaire on childhood motor skills. Am J Occup Ther 2000; 54: 484–93. 11006808 10.5014/ajot.54.5.484

[12] Driscoll DA, Budarf ML, Emanuel BS. A genetic etiology for DiGeorge syndrome: consistent deletions and microdeletions of 22q11. Am J Hum Genet 1992; 50: 924–33. 1349199 PMC1682598

[13] Niarchou M, Zammit S, van Goozen SH, Thapar A, Tierling HM, Owen MJ, Psychopathology and cognition in children with 22q11.2 deletion syndrome. Br J Psychiatry 2014; 204: 46–54. 24115343 10.1192/bjp.bp.113.132324 PMC3877833

[14] Schneider M, Debbané M, Bassett AS, Chow EWC, Fung WLA, Van Den Bree MBM, Psychiatric disorders from childhood to adulthood in 22q11.2 deletion syndrome: results from the international consortium on brain and behavior in 22q11.2 deletion syndrome. Am J Psychiatry 2014; 171: 627–39. 24577245 10.1176/appi.ajp.2013.13070864 PMC4285461

[15] Monks S, Niarchou M, Davies AR, Walters JT, Williams N, Owen MJ, Further evidence for high rates of schizophrenia in 22q11.2 deletion syndrome. Schizophr Res 2014; 153: 231–6. 24534796 10.1016/j.schres.2014.01.020

[16] Van Aken K, Swillen A, Beirinckx M, Janssens L, Caeyenberghs K, Smits-Engelsman Bouwien B. Prospective control abilities during visuo-manual tracking in children with 22q11.2 Deletion syndrome compared to age- and IQ-matched controls. Res Dev Disabil 2010; 31: 634–41. 20181458 10.1016/j.ridd.2010.01.002

[17] Van Aken K, De Smedt B, Van Roie A, Gewillig M, Devriendt K, Fryns JP, Motor development in school-aged children with 22q11 deletion (velocardiofacial/DiGeorge syndrome). Dev Med Child Neurol 2007; 49: 210–3. 17355478 10.1111/j.1469-8749.2007.00210.x

[18] Sobin C, Monk SH, Kiley-Brabeck K, Khuri J, Karayiorgou M. Neuromotor deficits in children with the 22q11 deletion syndrome. Mov Disord 2006; 21: 2082–9. 16991148 10.1002/mds.21103 PMC2753869

[19] Swillen A, Feys H, Adriaens T, Nelissen L, Mertens L, Gewillig M, Early motor development in young children with 22q.11 deletion syndrome and a conotruncal heart defect. Dev Med Child Neurol 2005; 47: 797–802. 16288668 10.1017/S0012162205001696

[20] Roizen NJ, Antshel KM, Fremont W, AbdulSabur N, Higgins AM, Shprintzen RJ, 22q11.2DS deletion syndrome: developmental milestones in infants and toddlers. J Dev Behav Pediatr 2007; 28: 119–24. 17435462 10.1097/01.DBP.0000267554.96081.12

[21] Bassett AS, McDonald-McGinn DM, Devriendt K, Digilio MC, Goldenberg P, Habel A, Practical guidelines for managing patients with 22q11.2 deletion syndrome. J Pediatr 2011; 159: 332–9.e1. 21570089 10.1016/j.jpeds.2011.02.039 PMC3197829

[22] Henderson S, Sugden D. The Movement Assessment Battery for Children (2nd edn). Pearson, 2007.

[23] Wilson BN, Crawford SG. The developmental coordination disorder questionnaire 2007. Phys Occup Ther Pediatr 2012; 29: 182–202. 10.1080/01942630902784761 19401931

[24] American Psychiatric Association. Diagnostic and Statistical Manual of Mental Disorder (5th edn) (DSM-5). APA, 2013.

[25] Wechsler D. WASI: Wechsler Abbreviated Scale of Intelligence. Psychological Corporation, 1999.

[26] Cambridge Cognition. *CANTAB Eclipse Test Administration Guide version 3.* Cambridge Cognitition, 2006.

[27] Heaton R, Chelune G, Talley J, Kay G, Curtiss G. Wisconsin Card Sort Test Manual: Revised and Expanded. Psychological Assessment Resources Inc., 1993.

[28] Rutter M, Bailey A, Lord C. Social Communication Questionnaire. Western Psychological Services, 2003.

[29] Angold A, Prendergast M, Cox A, Harrington R, Simonoff E, Rutter M. The Child and Adolescent Psychiatric Assessment (CAPA). Psychol Med 2009; 25: 739. 10.1017/s003329170003498x 7480451

[30] Tsiotra GD, Flouris AD, Koutedakis Y, Faught BE, Nevill AM, Lane AM, A comparison of developmental coordination disorder prevalence rates in Canadian and Greek children. J Adolesc Health 2006; 39: 125–7. 16781974 10.1016/j.jadohealth.2005.07.011

[31] Niarchou M, Martin J, Thapar A, Owen MJ, van den Bree MBM. The clinical presentation of attention deficit-hyperactivity disorder (ADHD) in children with 22q11.2 deletion syndrome. Am J Med Genet B Neuropsychiatr Genet 2015; 168: 730–8. 26400629 10.1002/ajmg.b.32378 PMC4737239

[32] Kopp S, Beckung E, Gillberg C. Developmental coordination disorder and other motor control problems in girls with autism spectrum disorder and/or attention-deficit/hyperactivity disorder. Res Dev Disabil 2010; 31: 350–61. 19910158 10.1016/j.ridd.2009.09.017

[33] Suwa H, Matsushima E, Ohta K, Mori K. Attention disorders in schizophrenia. Psychiatry Clin Neurosci 2004; 58: 249–56. 15149289 10.1111/j.1440-1819.2004.01227.x

[34] Schiffman J, Sorensen HJ, Maeda J, Mortensen EL, Victoroff J, Hayashi K, Childhood motor coordination and adult schizophrenia spectrum disorders. Am J Psychiatry 2009; 166: 1041–7. 19605535 10.1176/appi.ajp.2009.08091400 PMC3699872

[35] Schmahmann JD. Disorders of the cerebellum: ataxia, dysmetria of thought, and the cerebellar cognitive affective syndrome. J Neuropsychiatry Clin Neurosci 2004; 16: 367–78. 15377747 10.1176/jnp.16.3.367

[36] Bish JP, Pendyal A, Ding L, Ferrante H, Nguyen V, McDonald-McGinn D, Specific cerebellar reductions in children with chromosome 22q11.2 deletion syndrome. Neurosci Lett 2006; 399: 245–8. 16517069 10.1016/j.neulet.2006.02.001

[37] Van Amelsvoort T, Daly E, Robertson D, Suckling J, Ng V, Critchley H, Structural brain abnormalities associated with deletion at chromosome 22q11: Quantitative neuroimaging study of adults with velo-cardio-facial syndrome. Br J Psychiatry 2001; 178: 412–9. 11331556 10.1192/bjp.178.5.412

[38] Kates WR, Burnette CP, Bessette BA, Folley BS, Strunge L, Jabs EW, Frontal and caudate alterations in velocardiofacial syndrome (deletion at chromosome 22q11.2). J Child Neurol 2004; 19: 337–42. 15224707 10.1177/088307380401900506

[39] Sugama S, Bingham PM, Wang PP, Moss EM, Kobayashi H, Eto Y. Morphometry of the head of the caudate nucleus in patients with velocardiofacial syndrome (del 22q11.2). Acta Paediatr 2000; 89: 546–9. 10852189 10.1080/080352500750027826

[40] Eliez S, Barnea-Goraly N, Schmitt JE, Liu Y, Reiss AL. Increased basal ganglia volumes in velo-cardio-facial syndrome (deletion 22q11.2). Biol Psychiatry 2002; 52: 68–70. 12079732 10.1016/s0006-3223(02)01361-6

[41] Sieberer M, Haltenhof H, Haubitz B, Pabst B, Miller K, Garlipp P. Basal ganglia calcification and psychosis in 22q11.2 deletion syndrome. Eur Psychiatry 2005; 20: 567–9. 15967641 10.1016/j.eurpsy.2005.04.002

[42] Mok KY, Sheerin U, Simón-Sánchez J, Salaka A, Chester L, Escott-Price V, Deletions at 22q11.2 in idiopathic Parkinson's disease: a combined analysis of genome-wide association data. Lancet Neurol 2016; 15: 585–96. 27017469 10.1016/S1474-4422(16)00071-5 PMC4828586

[43] Butcher NJ, Kiehl T-R, Hazrati L-N, Chow EWC, Rogaeva E, Lang AE, Association between early-onset Parkinson disease and 22q11.2 deletion syndrome: identification of a novel genetic form of Parkinson disease and its clinical implications. JAMA Neurol 2013; 70: 1359–66. 24018986 10.1001/jamaneurol.2013.3646 PMC4464823

